# Clinical effects of atorvastatin combined with conbercept in the treatment of patients with macular edema secondary to retinal vein occlusion and carotid plaque: study protocol for a prospective randomized controlled trial

**DOI:** 10.1186/s13063-024-08082-0

**Published:** 2024-04-08

**Authors:** Bangtao Yao, Bei Wang, Jun Yang, Yan Geng, Hao Yu, Yuhui Liu, Gang Liu, Xiuying Wang

**Affiliations:** 1 Department of Ophthalmology, Nanjing Lishui People’s Hospital, Zhongda Hospital Lishui branch, Southeast University, Nanjing, Jiangsu Province China; 2 Department of Neurology, Nanjing Lishui People’s Hospital, Zhongda Hospital Lishui branch, Southeast University, Nanjing, Jiangsu Province China; 3 Department of Endocrinology, Nanjing Lishui People’s Hospital, Zhongda Hospital Lishui branch, Southeast University, Nanjing, Jiangsu Province China; 4 Department of Ophthalmology, Jiangsu Province Hospital, The First Affiliated Hospital of Nanjing Medical University, Nanjing, Jiangsu Province China

**Keywords:** Atorvastatin, Anti-vascular endothelial growth factor, Best-corrected visual acuity, Carotid plaques, Central subfield thickness, Intravitreal conbercept, Macular edema, Retinal vein occlusion

## Abstract

**Introduction:**

Intravitreal injections of anti-vascular endothelial growth factor (anti-VEGF) drugs have been widely used in patients with macular edema (ME) secondary to retinal vein occlusion (RVO); however, recurrence is a major concern. This study aims to observe the clinical effects of atorvastatin and intravitreal therapy in the treatment of patients with branch or central RVO-ME and coexistent carotid plaques (CP).

**Methods and analysis:**

A prospective randomized controlled clinical trial will be conducted. Sixty-four patients diagnosed with branch or central RVO-ME and coexistent CP will be enrolled and randomly allocated in a 1:1 ratio to the control and experimental groups. The control group will be treated with intravitreal conbercept monthly for 3 months, followed by monthly evaluation and injection of pro re nata (PRN) for 12 months, while the experimental group will be treated with oral atorvastatin 20 mg daily combined with the control group treatment. If a drop of best-corrected visual acuity (BCVA) is more than five Early Treatment Diabetic Retinopathy Study (ETDRS) letters (one line) or an increment in central subfield thickness (CSFT) of 100 μm (or a 10% increment from the previous visit), intravitreal re-treatment will be performed. Outcome measurements include CSFT, BCVA, number of injections, and incidence of adverse events during the 12-month follow-up period. Differences between groups will be evaluated using Student’s *t*-test, and comparisons between groups will be evaluated using repeated-measures analysis of variance.

**Ethics and dissemination:**

The study has been approved by the Institutional Review Board of Nanjing Lishui People’s Hospital, Nanjing, China (approval number 2023KY0418-12, dated 18 April 2023), and has been registered on chictr.org.cn. Written informed consent will be collected from each patient and the results of this trial will be submitted to a peer-reviewed journal.

**Trial registration:**

Chinese Clinical Trial Registry ChiCTR2300071359. Registered on 12 May 2023.

**Supplementary Information:**

The online version contains supplementary material available at 10.1186/s13063-024-08082-0.

## Introduction

Retinal vein occlusion (RVO) is the second most common retinal vascular disease and is characterized by a sudden painless visual reduction [1, 2]. However, the exact mechanisms underlying RVO remain unclear. Recent evidence has demonstrated that RVO is closely associated with carotid plaques (CP), an important risk factor for stroke [3]. Evidence has confirmed that CP is observed in 54.3% and 76.7% of patients with RVO and branch RVO, respectively [3, 4]. Retinal arteriosclerosis and thrombosis caused by CP can result in retinal venous reflux disorders and secondary RVO [3–5]. RVO can be a predictor of CP and should be evaluated using carotid Doppler ultrasound [1]. Carotid Doppler ultrasound is non-invasive, useful for screening and diagnosing CP, and can accurately evaluate carotid intima-media thickness [3]. However, it is not commonly used for the clinical treatment of RVO.

Macular edema (ME) is the most severe and frequent complication of RVO [2]. Intravitreal injections of anti-vascular endothelial growth factor (anti-VEGF) drugs (e.g., ranibizumab, aflibercept, and conbercept) have been widely used as first-line treatment for patients with RVO-ME [2, 6–8]. However, they have several limitations. First, the clinical effect of anti-VEGF drugs is not long-lasting, and the recurrence of ME indicates repeated injections. Hunt et al. reported that the median number of injections of ranibizumab and aflibercept for branch RVO-ME was seven over 12 months [2]. Sun et al. used conbercept to treat RVO-ME and the mean number of injections was 7.14 ± 1.90 in branch RVO and 7.59 ± 1.39 in central RVO, over 9 months [8]. Second, frequent injections increase surgical risks, including endophthalmitis and secondary glaucoma [6]. Third, anti-VEGF drugs are expensive, and reinjections lead to an economic burden for patients. Therefore, recurrence during follow-up is a major concern for ophthalmologists [7]. Risk factors such as retinal structures (e.g., elevated central retinal thickness and disorganization of retinal inner layers) have been described; however, to the best of our knowledge, investigations of systemic factors such as CP in RVO-ME have not been discussed in the “EURETINA Guidelines for the Diagnosis and Treatment of Retinal Vein Occlusion” [9].

Clinical trials have shown that regular statin use plays a significant role in preventing cerebrovascular atherosclerosis and reducing the risk of cerebrovascular accidents [10]. Atorvastatin, a statin (lipid-lowering drug), has proven to be effective in stabilizing and reversing plaques in patients with carotid atherosclerosis in combination with antiplatelet agents [11–13]. Furthermore, a multicentre study showed that atorvastatin significantly reduced the risk of cardiovascular and cerebrovascular accidents in patients with CP, and the risks of stroke and coronary artery accidents decreased by 33% and 43%, respectively [14].

Recently, atorvastatin has been proven useful in reducing retinal VEGF expression and inhibiting inflammation [15, 16]. Oral atorvastatin was effective in patients with diabetic ME and age-related macular degeneration [17, 18]. Similarly, intravitreal statins can help reduce the development of proliferative vitreoretinopathy [19]. However, clinical investigations of atorvastatin in the treatment of RVO-ME and CP in the current studies are lacking.

## Methods and analysis

### Study objectives

This study aims to observe the clinical effects of atorvastatin and intravitreal therapy in the treatment of patients with branch or central RVO-ME and coexistent CP.

### Trial design

In this trial, 64 patients diagnosed with branch or central RVO-ME and coexistent CP will be enrolled and randomly allocated in a 1:1 ratio to the control and experimental groups. The control group will be treated with intravitreal conbercept (Chengdu Kanghong Pharmaceutical; National Drug Approval No. S20130012), using the 3+ pro re nata (PRN) scheme (monthly for 3 months, followed by monthly evaluation and injection PRN for 12 months) and the experimental group will be treated with oral atorvastatin (Beijing Jialin Pharmaceutical Co., Ltd., GYZZH20093819) 20 mg daily combined with the control group treatment.

### Eligibility criteria

Patients diagnosed with RVO-ME and CP are based on indirect ophthalmoscopy, fundus fluorescein angiography (FFA), spectral-domain optical coherence tomography (SD-OCT), and carotid Doppler ultrasound.

### Inclusion criteria

The inclusion criteria are as follows: (1) patients with a branch or central RVO, aged between 45 and 75 years old; (2) central sub-field thickness (CSFT) > 250 μm, best corrected visual acuity (BCVA) < 0.5; and (3) body mass index < 28 kg/m^2^, carotid stenosis without recanalization surgery.

### Exclusion criteria

The exclusion criteria are as follows: (1) patients with severe cataract, macular membrane, optic neuropathy, glaucoma, and uveitis; (2) patients with a branch or central RVO-ME in both eyes; (3) patients with macular edema secondary to age-related macular degeneration, diabetic retinopathy, Coats disease, Eales disease, or high myopia; (4) history of intravitreal injection, retinal surgery, laser photocoagulation, or ocular trauma; (5) history of taking lipid-lowering drugs in the past month; (6) patients with severe cardiovascular, cerebrovascular, liver, and kidney dysfunction; and (7) patients who cannot tolerate surgery or have poor compliance.

### Withdrawal criteria

The withdrawal criteria are as follows: (1) the follow-up was lost; (2) informed consent was withdrawn; and (3) decision to withdraw for severe adverse events.

### Methodology

**Fig. 1:**
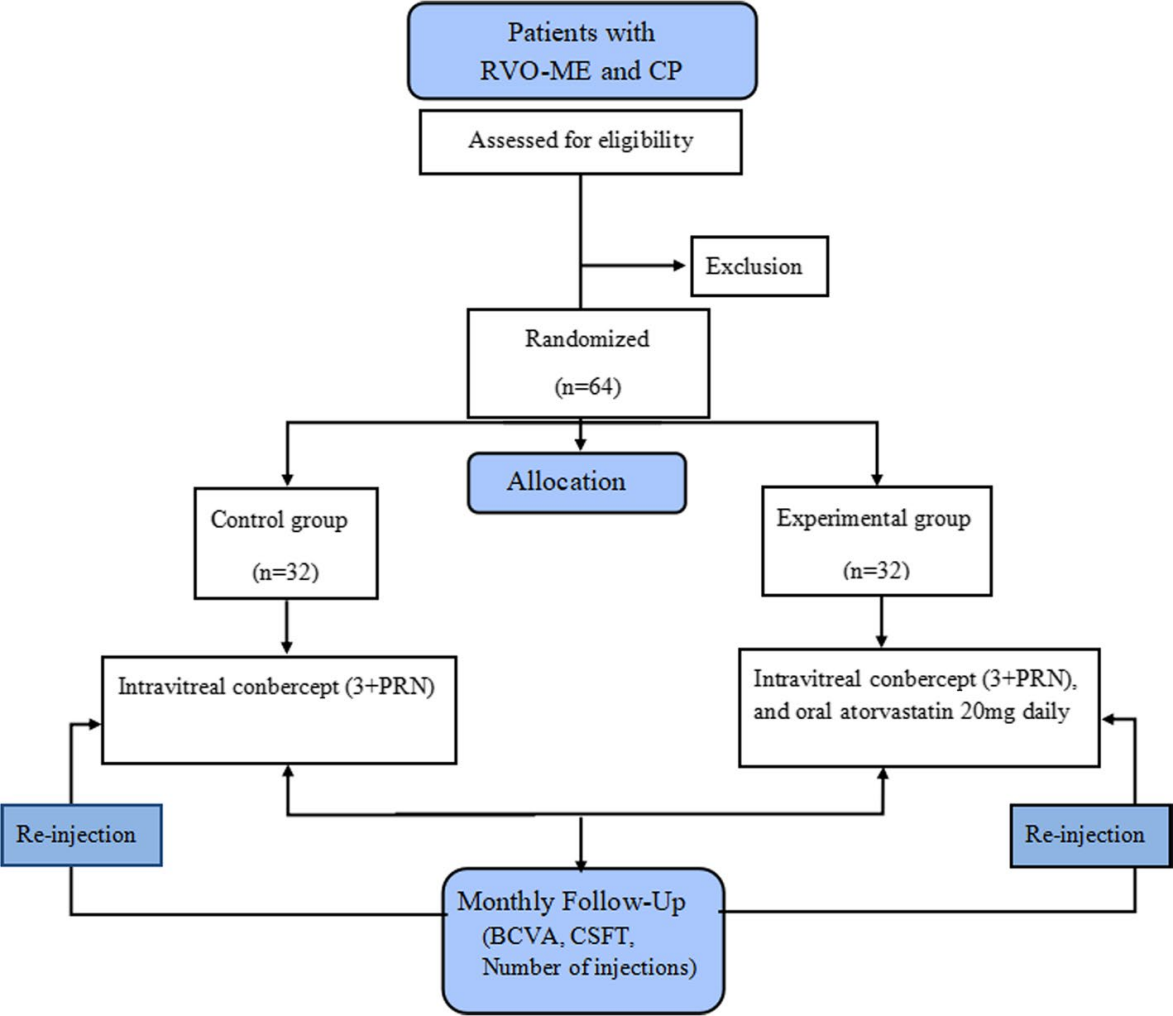
Study design. PRN, pro re nata; RVO, retinal vein occlusion; ME, macular edema; CP, carotid plaque; BCVA, best corrected visual acuity; CSFT, central subfield thickness

A prospective, randomized controlled clinical trial will be conducted at Nanjing Lishui People’s Hospital. Patients diagnosed with branch or central RVO-ME and CP will be enrolled in this study. They will be randomly allocated in a 1:1 ratio to the control and experimental groups. The study design is illustrated in Fig. 1.

### Randomization

Patients will be randomly divided into two groups in a 1:1 ratio using SPSS (Statistical Package for Social Sciences, version 26.0, IBM, Armonk, NY, USA). An independent investigator will generate a random sequence and allocate interventions.

### Masking

Patients and clinicians will be blinded after assignment. The implementation and maintenance of the randomization and masking methods will be completely validated. To avoid evaluation bias, the treatment groups will be hidden from the ophthalmologist who collects the clinical data during each follow-up period. Analysts dealing with the data will also be masked.

### Interventions

All patients will undergo preoperative and postoperative investigations. Systemic examinations include routine blood tests, blood biochemistry, infection measurements (HIV, hepatitis B and C, and syphilis), chest computed tomography (CT), electrocardiography, and carotid Doppler ultrasound. Ophthalmic examinations include BCVA, slit-lamp, intraocular pressure, indirect ophthalmoscopy, FFA, and SD-OCT. The timeline of data collection is shown in Table 1.

**Table 1:** Outcome measurements and data collection at each follow-up timepoint

Phase	Screen	Postoperative follow-up											
Follow-up	0	1	2	3	4	5	6	7	8	9	10	11	12
Time points	Before operation	1 month after operation	2 months after operation	3 months after operation	4 months after operation	5 months after operation	6 months after operation	7 months after operation	8 months after operation	9 months after operation	10 months after operation	11 months after operation	12 months after operation
Informed consent	●												
Inclusion and exclusion criteria	●												
Medical history collection	●												
Ophthalmic examinations													
BCVA	●	●	●	●	●	●	●	●	●	●	●	●	●
Slit lamp	●	●	●	●	●	●	●	●	●	●	●	●	●
Indirect ophthalmoscopy	●	●	●	●	●	●	●	●	●	●	●	●	●
IOP	●	●	●	●	●	●	●	●	●	●	●	●	●
SD-OCT	●	●	●	●	●	●	●	●	●	●	●	●	●
FFA	●						●						●
Laboratory examinations													
Routine blood test	●						●						●
Blood biochemistry	●			●			●			●			●
Infection measurements	●												●
Carotid doppler ultrasound	●			●			●			●			●
Electrocardiogram	●						●						●
Chest CT	●												●
Adverse events recorded	●	●	●	●	●	●	●	●	●	●	●	●	●

*BCVA* best corrected visual acuity, *IOP* intraocular pressure, *SD-OCT* spectral-domain optical coherence tomography, *FFA* fluorescence fundus angiography, *CT* computed tomography

### Outcome measurements

Monthly follow-ups will be performed after surgery in both groups. The necessary investigations will be conducted at each follow-up visit.

The outcome measurements include CSFT, BCVA, number of injections, and incidence of adverse events.

### Primary outcomes

The primary outcome is the average change in CSFT from baseline to 12 months.

### Secondary outcomes

Secondary outcomes are BCVA, average number of injections, and incidence of adverse events at each visit during the 12-month follow-up visit.

### Safety and combined operation-related adverse events

Possible operation-related complications include elevated intraocular pressure, endophthalmitis, traumatic cataract, vitreous hemorrhage, retinal hemorrhage, retinal detachment, and drug-related adverse events (e.g., liver function damage and gastrointestinal discomfort). In this trial, all the above adverse events and possible causes will be recorded and managed accordingly.

### Follow-up plan

Monthly follow-ups and necessary re-examinations after surgery will be conducted. BCVA, slit lamp, intraocular pressure, indirect ophthalmoscopy, and SD-OCT will be performed at each visit. Blood biochemistry and carotid Doppler ultrasonography will be performed every 3 months. Routine blood tests, FFA, and electrocardiograms will be performed every 6 months. Infection measurements (HIV, hepatitis B and C, and syphilis) and chest CT will be performed during the last visit. The number of injections and the incidence of complications will be recorded and evaluated at each follow-up.

### Re-injection criteria

If the BCVA drops by more than five Early Treatment Diabetic Retinopathy Study (ETDRS) letters (one line) or an increment in CSFT of 100 μm (or a 10% increment from the previous visit), intravitreal re-treatment will be performed.

### Sample size calculation

The sample size is estimated as follows: according to the results from our pre-experiment, the average CSFT in the control and experimental groups was 263.72 ± 30.63 μm and 241.17 ± 18.54 μm, respectively. The type I error rate (*β*) was set at 0.05, with 80% power at 5% significance and 1:1 randomization. *Zα* = 1.96, *Zβ* = 0.84, standard deviation (*σ*) in experimental group = 30.63, difference in means (*δ*) = 263.72 − 241.17 = 22.55.

Sample size $$n=\frac{2{\left({z}_a+{z}_{\beta}\right)}^{2\ast }{\sigma}^2}{\delta^2}=2\times {\left(1.96+0.84\right)}^2\times 30.{63}^2/22.{55}^2=29.$$

Thus, 29 patients will be required in each group. Considering a loss ratio of 10%, the total sample size will increase to 32 patients in each group.

### Statistical analysis

Data from the patients’ clinical records will be processed using SPSS. For normally distributed data, continuous variables will be expressed as mean ± standard deviation, difference between groups will be evaluated by the Student’s *t*-test, and comparisons inter-group at each endpoint will be evaluated by repeated measures analysis of variance. The Bonferroni method will be used to adjust the overall significance level for multiplicity. For abnormally distributed data, continuous variables will be expressed as M (Q1, Q3), differences between groups will be evaluated using the Mann–Whitney *U* test, and comparisons between groups at each endpoint will be evaluated using a generalized linear mixed model. Categorical variables will be expressed as counts (%), and the chi-square test will be performed. The effect of missing data on the trial results will be assessed using sensitivity analyses of the augmented datasets. Dropouts will be included in the analysis by using modern imputation methods for missing data. The BCVA will be converted to the minimum resolution angle in logarithmic form before data analysis. The clinical data of the branch and central RVO will be sub-analyzed. Two-tailed tests of significance will be performed, and *P*-values < 0.05 will be considered statistically significant.

### Provisions for post-trial care

There is no anticipated harm and compensation for trial participation.

### Ethics and dissemination

The study has been approved by the institutional review board of Nanjing Lishui People’s Hospital, Nanjing, China (approval number 2023KY0418-12, dated 18 April 2023) and registered with the Chinese clinical trial registry (http://www.chictr.org.cn/, No. ChiCTR2300071359). Written informed consent will be collected from each patient. They will be informed about the study procedures, possible treatment risks, and their right to withdraw from the trial. The results of this trial will be submitted to a peer-reviewed journal.

### Access to the full protocol, participant-level dataset and statistical code

The datasets analyzed in the current study and the statistical code are available from the corresponding author upon reasonable request, as is the full protocol.

### Patient public involvement

The patients and the public were not involved in the design, conduct, reporting, or dissemination of our research plans.

## Discussion

This is a prospective, randomized controlled clinical trial investigating the clinical effect of atorvastatin and intravitreal conbercept in the treatment of ME in patients with a branch or central RVO-ME and coexistent CP. We speculate that the treatments in the experimental group may have significantly improved the carotid intima-media thickness and inhibited VEGF and inflammation in the retina. This study may provide an opportunity to reduce the recurrence rate of RVO-ME. Further studies with larger sample sizes and more data are required to better understand the effects of combined treatments.

### Oversight and monitoring

#### Composition of the coordinating center and trial steering committee

The trial steering committee will be formed by the principal investigator and the co-investigators. The committee will manage the entire project and submit a report for publication at the end of the study. The committee will appoint inspectors of the Project Management Group once a month. The project management group will oversee standardization in line with clinical practice requirements and submit their reports to the trial steering committee.

#### Composition of the data monitoring committee, its role and reporting structure

A member will be designated by the principal investigator to handle the data. Electronic paper case report forms and management tools will be used to collect the data. The trial steering committee managed the trial under the supervision of the project management group.

### Frequency and plans for auditing trial conduct

The project management group will meet every 2 weeks to review the trial. The Trial Steering Group and Ethics Committee will monitor throughout the trial period. Owing to the low-risk nature of the intervention, an independent Data Monitoring Committee will not be considered.

### Protocol amendments

If there are any changes to the protocol, the sponsor and funder will be notified first, the principal investigator will notify the centers, and a copy of the revised protocol will be sent to the principal investigator to be added to the investigator-site file. Any deviations from the protocol will be fully documented using a breach report. This protocol will be updated in the clinical trial registry.

### Trial status

At the time of manuscript submission, this trial has recruited 14 patients. This trial will be completed by December 2024. The current protocol (Code: ChiCTR2300071359) is version 1.0, dated 18 April 2023.

### Supplementary Information

**Supplementary Material 1.**

## Data Availability

The datasets used and/or analyzed during the current study are available from the corresponding author on reasonable request.

## Abbreviations

BCVA: Best corrected visual acuity
IOP: Intraocular pressure
SD-OCT: Spectral-domain optical coherence tomography
FFA: Fluorescence fundus angiography
CT: Computed tomography
RVO: Retinal vein occlusion
ME: Macular edema
VEGF: Vascular endothelial growth factor
CP: Carotid plaque
CSFT: Central subfield thickness
ETDRS: Early Treatment Diabetic Retinopathy Study
